# Supplementary material for: The COVID-19 Pandemic Vulnerability Index (PVI) Dashboard: Monitoring county-level vulnerability using visualization, statistical modeling, and machine learning
Source: medRxiv. 2020 Sep 13:2020.08.10.20169649. Originally published 2020 Aug 13. Preprint. [Version 3] doi: 10.1101/2020.08.10.20169649 (PMC7430608; doi:10.1101/2020.08.10.20169649)
Supplement: Supplement 2020 [file 96638-2020.08.10.20169649-1.pdf]

Supplemental Materials

Details of Dashboard Features

Dynamic documentation is available via links from the Dashboard landing page (https://covid19pvi.niehs.nih.gov/) in the form of a Quick Start Guide for users (https://www.niehs.nih.gov/research/programs/coronavirus/covid19pvi) and a Details page (https://www.niehs.nih.gov/research/programs/coronavirus/covid19pvi/details/index.cfm). Here, we summarize features of the current Dashboard interface, depicted in Figure S2.

On loading, the Dashboard displays a map of the continental United States, overlaid with the top 250 PVI profiles (by rank) for the current day. Users can navigate the map by dragging and/or by zooming in and out with the PLUS/MINUS icons or keyboard shortcuts. Clicking on a county brings up the current daily scorecard. The scorecard shows the graphic representation of the PVI, the overall score, and the rank and score for each data stream. Selection of a county also populates the surrounding panels with county-specific information. Scrollable panels on the left of the Dashboard include plots of vulnerability drivers relative to their nationwide distribution across all U.S. counties, with the location of the selected county delineated. The panels across the bottom of the Dashboard report cumulative county numbers of cases and deaths; timelines of cumulative cases, deaths, PVI scores, and PVI ranks; daily changes in cases and deaths for the most recent 14-day period (a measure commonly used in reopening guidelines); and predicted cases and deaths for a seven-day forecast horizon.

The main menu bar provides numerous options for visualizing the map that are detailed in the Quick Start Guide. The base map can be changed from the default gray to options that include satellite imagery and topology (Change Map menu option). Users can choose a WebMap by inserting a WebMap Portal Item ID. Details of the WebMap Extent options are available through ESRI (ESRI 2020). Cumulative case and death counts are plotted as a default map layer, which can be changed or removed through the COVID-19 Legend menu option. The size and opacity of the PVIs displayed on the map can be changed through the PVI Model Legend option. By default, the Dashboard displays the case and death counts from the Johns Hopkins Dashboard and the PVI model from the current day. Interactive options to display data from different days are available in the 'Covid-19 Layers' panel for cumulative case and death data and 'PVI Model Layer' panel for the historical PVI.

To generate the display in Figure S2, the 'Quick Filter' was used to select all counties in the state of Alabama. The county of Autauga was highlighted by mouse-click, bringing up the Scorecard and populating info panels along the left and bottom of the Dashboard. Scrolling down the left side under 'Quick Filter' displays the PVI slice legend and histograms of the overall and slice-wise PVI across all U.S. counties. For each histogram, a black line indicates the location of the selected county within this distribution. For all distributions, lower values (to the left) indicate lower relative vulnerability (i.e. shorter slices) whereas higher values (to the right) reflect higher vulnerability (i.e. longer slices).

In addition to the Change Map, PVI Model Legend, and Covid-19 Legend options, additional display options are available through the main drop-down menu (see the Quick Start

Guide). The PVI Model Filter option allows the interactive filtering of display options, including the number of profiles displayed. The top-ranked counties, which are those with the highest vulnerability according to the overall PVI, are displayed by default. The PVI Model Filter option also allows users to display the most vulnerable counties based on individual data streams, with options for multi-level filters. These options for restricting the ranges of one or more slices enabling the identification of counties with similar profiles (see Main Text description of Figure 3 for an example application). The PVI Model Clusters option provides additional opportunities for data-driven contextualization. The two options for clustering PVI profiles are labeled KMeans (agglomerative with k=10) and HClust (divisive, displaying the top-10 splits), after the algorithm used. Both options identify counties with a similar PVI profile "shape". The distance matrix is calculated from the integrated profile, which considers all slice scores and weights. For KMeans, the clustering is implemented as a Java port of the *kmeans* R function using the Hartigan-Wong algorithm (R Core Team 2018). For HClust, the clustering is implemented as a Java port of the *hclust* R function using the "complete" method (R Core Team 2018). By filtering the Dashboard to display only specific clusters, users can identify clusters with multivariate profile similarity. This enables the detection of clusters that may be geographically adjacent as well as geographically distinct to reflect the diversity of risk profiles on a national scale. The existence of geographically disparate areas with similar drivers highlights the need for integrated profiles to encourage effective local-level policies targeted at communities at risk.

Forecasting

To predict COVID-19 case and death counts, we model the observed log-counts for new cases and deaths in each U.S. county using a Bayesian functional model. We assume deaths are a proportion of active COVID cases, and we jointly model both cases and deaths so these data are shared between these observations. In this model, the expected case count is the case rate offset by the observed number of deaths and is spatially modeled across the United States. Currently, the model assumes a normal distribution on the logs of the observed counts.

Let C_{tj} and D_{tj} be the observed case and death count for day t, $1 \leq t \leq T$, and county j, $1 \leq j \leq J$. For county j, we also observe a vector $x_j = \left(1, x_{1j}, \ldots, x_{mj}\right)'$ of m explanatory variables that are static over time (e.g., population density) and a vector $z_{tj} = \left(z_{1tj}, \ldots, z_{ktj}\right)'$ of k explanatory variables that are observed each day (e.g., social distancing metrics). Let X_j be the matrix of observed explanatory variables, both static and dynamic, for the T time points in county j. Conditional on knowing the case and death rates λ_{Cij} and λ_{Dij} , we assume that C_{ij} and D_{ij} are Poisson variates where

$$\lambda_{ctj} = exp(X_j\beta + \gamma_c(s_j, t) + \log[\bar{C}_{tj}] + \epsilon_{tj}^{C}), \qquad (1)$$

and

$$\lambda_{Dtj} = exp\left(X_j\alpha + \gamma_D(s_j, t) + \log[\bar{\lambda}_{ctj}] + \epsilon_{tj}^{D}\right). \quad (2)$$

To define these rates, log $[\bar{C}_{ij}]$ is the log geometric-mean of the previously observed count; log $[\bar{\lambda}_{ctj}]$ is the log of the new case rate for the last m days (i.e., log $[\bar{\lambda}_{ctj}] = \frac{1}{m}\sum_{i=t-m}^{t} X_j\beta + \gamma_c(s_j, i) + \bar{C}_{ij}$); $\mu_c(s_j, t)$ and $\mu_D(s_j, t)$ are spatial process accounting for unobserved heterogeneity in the response at time t and county location s_j; and $\epsilon_{tj}^{C} \sim N(0, \tau_{cj}^{-1})$ and $\epsilon_{tj}^{D} \sim N(0, \tau_{Dj}^{-1})$. This defines a Poisson-lognormal model over C_{ij} and D_{ij}, which is an over-dispersed count model that allows for efficient sampling using Bayesian computation with conditional Gibbs updates.

The random fields borrow information from nearby counties under the assumption that geographically proximate counties have public health departments with similar testing strategies and testing resources and likely have similar responses to the pandemic, which would account for heterogeneity not captured by the covariates. Time is included as testing strategies and testing resources are expected to change over time as well as by region.

Modeling the Spatial-Temporal Power Term

Let $\gamma_C(s_j, t) = f_C(s_j)g_C(t)$ and $\gamma_D(s_j, t) = f_D(s_j)g_D(t)$, where $f_C \sim GP(0, \sigma_C[\cdot,\cdot])$ and $f_D \sim GP(0, \sigma_D[\cdot,\cdot])$, which are Gaussian processes (Rasmussen & Williams 2006)with a 0 mean and squared exponential covariance kernel functions $\sigma_C[s, s'] = \exp[-\tau_c(s - s')^2]$ and $\sigma_C[s, s'] = \exp[-\tau_D(s - s')^2]$, where τ_c and $\tau_D$are length-scale parameters controlling the amount of correlation between spatial locations s and s'. For f_C and f_D, it is generally assumed that the covariance kernel has an unknown variance component. In our case, we fix this parameter to 1 because it is unidentifiable given g_c and g_D.

For g_c and g_D, we use first-order B-splines (De Boor 2001), which are local linear piecewise splines. That is,

$$g_c(t) = \sum_{k=1}^{K_c} \zeta_{ck} b_{ck}(t),$$

and

$$g_D(t) = \sum_{k=1}^{K_D} \zeta_{Dk} b_{Dk}(t),$$

where $b_{ck}(t)$ and $b_{Dk}(t)$ are defined on K_c and K_D evenly spaced knots, respectively. We chose linear splines to minimize end-knot variability. This formulation is a tensor product formulation (De Boor 2001; Wheeler 2019), which allows modeling the three-dimensional surface as the product of a two-dimensional surface and a one-dimensional surface.

Bayesian Specification and Computation

We conducted inference and prediction using Bayes' rule. As such, all parameters in the models described in Equations (1) and (2) are given proper priors, and inference is completed using Markov chain Monte Carlo (MCMC) methods. All coefficients on the explanatory covariates are given normal(0,10) priors. The precision terms, namely τ_{Cj}^{-1} and τ_{Dj}^{-1} , are given Gamma(10,1) priors. For the Gaussian processes, the length-scale terms τ_c and τ_D are given discrete uniform priors over a range of equally spaced values that cover a variety of covariance

functions. Finally, the ζ_{ck} and ζ_{Dk} terms, which specify the spline coefficients over the time component in the tensor product, are given Bayesian P-spline priors (Lang & Brezger 2004). This allows flexible smooth modeling of the time component and defines the variance of the tensor product Gaussian processes, conditional on the time component.

All priors are conditionally conjugate, with inference conducted using Gibbs sampling. In total, 11,000 MCMC samples were taken, with the first 1,000 disregarded as burn-in. We examined trace plots for convergence and mixing from separate chains. These indicated convergence in the chain, typically within 100 iterations, and reasonable mixing. We took posterior predictive observations (i.e., future cases and deaths) every 20th sample, for a total of 500 posterior predictive observations. Dashboard predictions are made by taking the mean and standard deviation of these observations. Because 1 is added to the original count, 1 is subtracted from the estimate. In the rare case that the estimated data point is negative, a 0 average case count or death count is recorded. Otherwise, non-integer values are used for the forecast.

A major issue involved in this construction is the dramatic increase in the computational time required for the Gaussian processes with more observations (i.e., algorithms on covariance matrices are intrinsically $O(n^3)$). Bayesian computation using the exact Gaussian process is not feasible for 3,142 distinct geographical locations, so, as an alternative, we use the method developed by Moran and Wheeler (2020), which employs compressed covariance matrices that are nearly exact to the original covariance matrix (i.e., constructed such that the max norm of the two matrices is approximately 1e-14), but has the added benefit of the computational complexity of $O(nlog^2n)$. Utilizing these algorithms, the computational time required is decreased by a factor of 40, from two weeks to 6 to 8 hours. Unlike Moran and Wheeler, we do not use Ambikasaran and Darve's (2013) HODLR compression technique due to its inability to scale to more than one dimension. Instead, we utilize Börm's (2010) H2 matrix compression method that utilizes the H2lib matrix library.

While the model produces reliable estimates, rapid increases in infection rate can occasionally generate outlier extrapolations. For reporting within the Dashboard, counties with < 100 cases were examined for the largest count increase week over week nationwide and multiplied by 1.5 a single time to derive a cases increase threshold. For counties with the prior week of actuals < 100, if the predicted cases from the afore mentioned Bayesian models were capped at this value. For counties with cases >= 100 in the prior period, no such threshold was applied. Similar logic was applied to deaths < 5 counts.

References

Ambikasaran, S. and E. Darve, An O(NlogN) Fast Direct Solver for Partial Hierarchically Semi-Separable Matrices. J Sci Comput 57(3):477-501, https://doi.org/10.1007/s10915-013-9714-z.

Borm S. 2020. Efficient numerical methods for non-local operators: H2-matrix compression, algorithms and analysis. Zurich: European Mathematical Society.

Centers for Disease Control and Prevention (CDC). 2020. Reopening Guidance for cleaning and disinfecting public spaces, workplaces, businesses, schools, and homes. https://www.cdc.gov/coronavirus/2019-ncov/community/reopen-guidance.html [accessed on 31 August 2020].

De Boor C. 1978. A practical guide to splines. Applied Mathematical Sciences, vol. 27. New York: Springer.

Esri. 2020. ArcGIS Javascript API, version 4.13. https://www.esri.com/en-us/home [accessed on 31 August 2020].

Lang S, Brezger A. 2004. Bayesian P-splines. J Comput Graph Stat 13(1):183-212, https://doi.org/10.1198/1061860043010.

Moran KR, Wheeler MW. 2020. Fast increased fidelity approximate Gibbs samplers for Bayesian Gaussian process regression. arXiv:2006.06537.

R Core Team. *R: A language and environment for statistical computing*. 2019, R Foundation for Statistical Computing: Vienna, Austria. https://www.R-project.org/ [accessed on 31 August 2020].

Rasmussen CE, Williams CKI. 2006. Gaussian processes for machine learning, vol. 2 Cambridge, MA: MIT Press.

Unacast. 2020. Social Distancing Scoreboard. https://www.unacast.com/covid19/social-distancing-scoreboard [accessed on 31 August 2020].

Wheeler MW. 2019. Bayesian additive adaptive basis tensor product models for modeling high dimensional surfaces: an application to high-throughput toxicity testing. Biometrics **75**(1):193-201, PMID: 30081432, https://doi:10.1111/biom.12942.

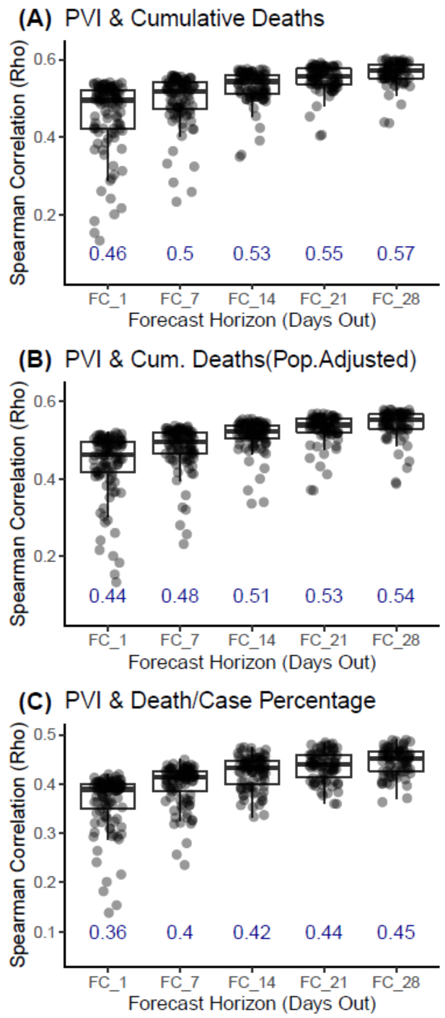

Figure S1.

Spearman correlation (Rho) estimates of daily PVI (by county) vs. cumulative deaths (A), population-adjusted cumulative deaths (B), and the case fatality rate (C) for 1, 7, 14, 21, or 28 days into the future. Mean Rho values for each time horizon are displayed in blue.

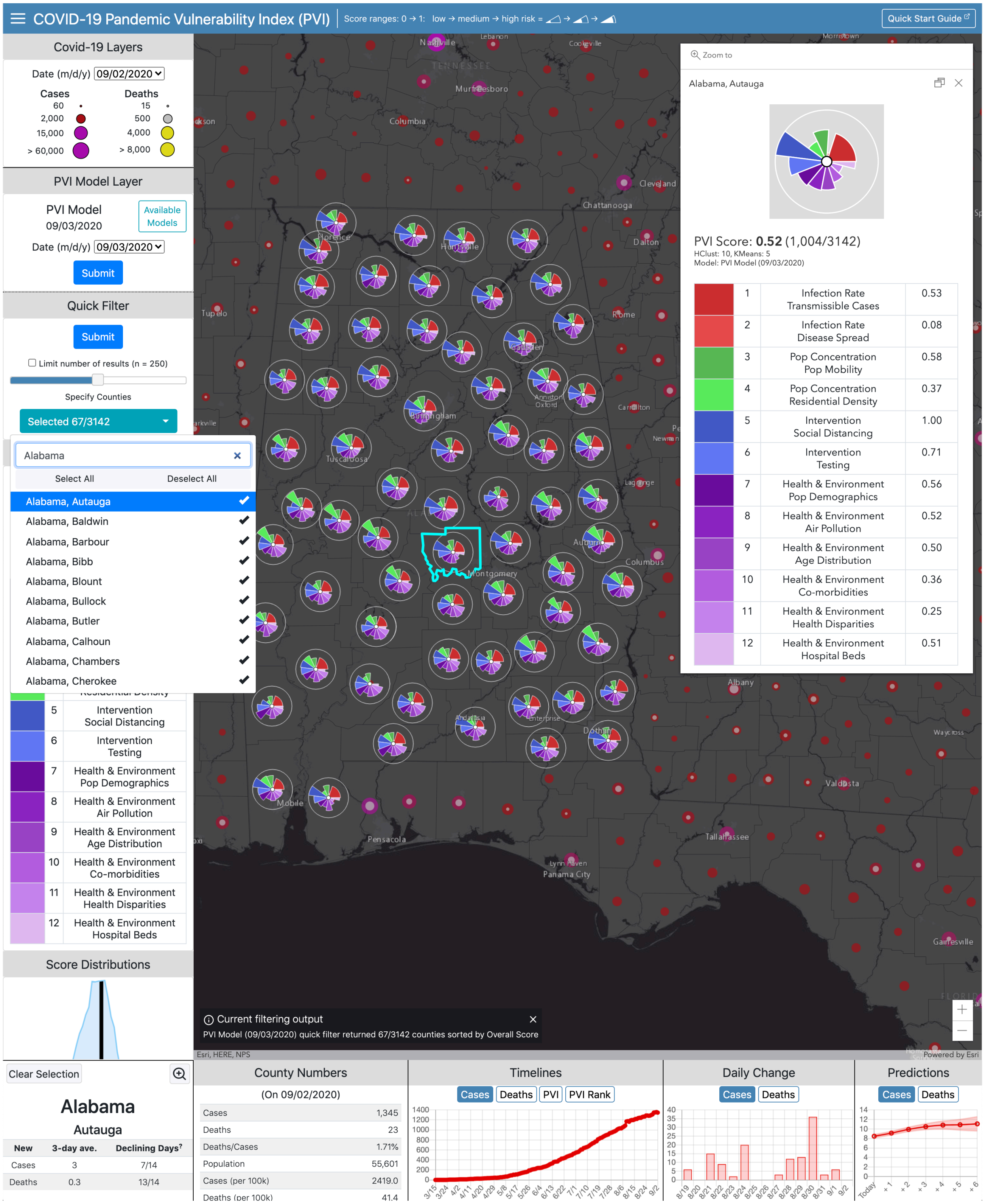

Figure S2.

A screenshot of the current Dashboard. The text provides detailed descriptions of the panels and menu options. This information is also available in the Quick Start Guide at https://www.niehs.nih.gov/research/programs/coronavirus/covid19pvi/index.cfm

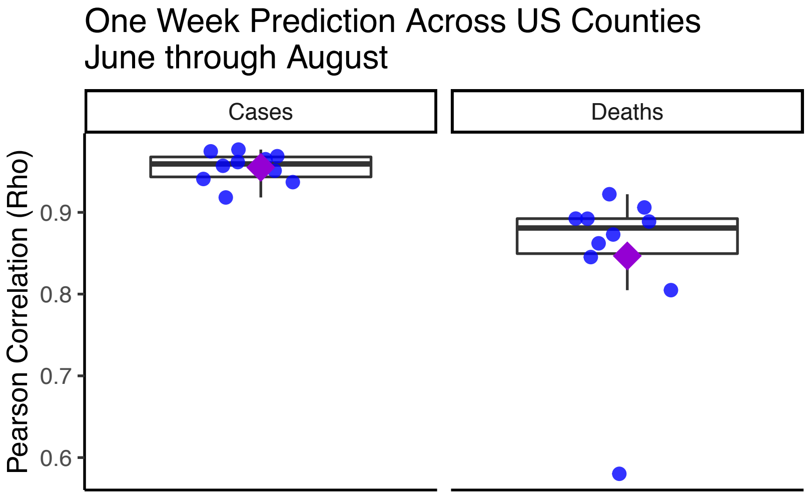

Figure S3.

Distributions of county level weekly prediction correlations (1 weekforecast horizons) for both Covid19 cases and deaths between June 3 and August 24, 2020 are displayed. Average correlations for the points are plotted as purple triangles.

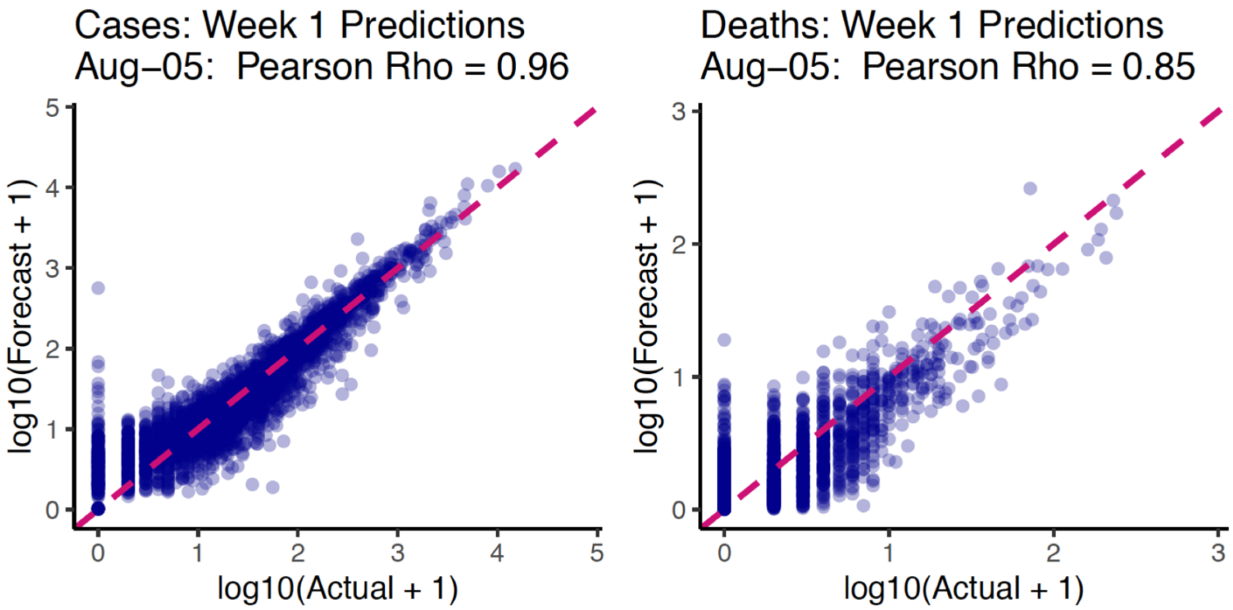

Figure S4.

Scatterplots of forecast versus actual (observed) Cases and Deaths for all counties for the week of August 5, 2020.

Table S1.

Datasets comprising the current PVI model. Note that this table is available with live links on the ‘Details’ page at: https://www.niehs.nih.gov/research/programs/coronavirus/covid19pvi/details/

Data Domain (% weight)<br>Data Slice (% weight)<br>Component(s)	Update Freq.		Description/Rationale	Source(s)
**Infection Rate (24%)**				
**Transmissible Cases (20%)**				
	Daily		*Population size divided by cases from the last 14 days.* Because of the 14-day incubation period, the cases identified in this period are the most likely to be transmissible. This metric is the number of “contagious” individuals relative to the population, so a higher number indicates a greater likelihood of the continued spread of infection.	Johns Hopkins University
**Disease Spread (4%)**				
	Daily		*Fraction of total cases from the last 14 days (one incubation period).* Because COVID-19 is thought to have an incubation period of up to 14 days, only a sustained decline in new infections over two weeks is sufficient to signal a reduction in the infection spread. This metric is always between 0 and 1, with values near 1 during the exponential growth phase and declining linearly to 0 over 14 days if there are no new infections.	Johns Hopkins University
**Population Concentration (16%)**				
**Population Mobility (8%)**				
**Daytime Population Density**	Static		*Estimated daytime population.* Higher daytime population density is expected to increase the spread of infection because more people are in closer proximity to each other.	The field “DPOPDENSCY” (2019 Daytime Pop Density) from the ESRI demographic analysis of American Community Survey data. 2018 CDC Social Vulnerability Index (adjunct variable)
**Baseline Traffic**	Static		*Average traffic volume per meter of major roadways in the county from 2018 EPA EJSCREEN.* Higher traffic volume is expected	2020 County Health Rankings

Data Domain (% weight)<br>Data Slice (% weight)<br>Component(s)	Update Freq.		Description/Rationale	Source(s)
			to increase the spread of infection due to more movement and interaction.	
**Residential Density (8%)**				
**Residential Density**	Static		*Integrates data from the 2014-2018 ACS on families in multi-unit structures, mobile homes, over-crowding (more people than rooms), being without a vehicle, and persons in institutionalized group quarters.* All of these variables are associated with higher residential density, which is expected to increase the spread of infection because more people are in closer proximity to each other.	2018 CDC Social Vulnerability Index (SVI Housing Type and Transportation Theme)
**Intervention Measures (16%)**				
**Social Distancing (8%)**				
	Daily		*A Unacast social distancing scoreboard grade is assigned by examining the change in overall distance traveled and the change in nonessential visits relative to a baseline (the same period during the previous year), based on cell phone mobility data.* The grade is converted to a numerical score, with higher values, meaning less social distancing (worse score), expected to increase the spread of infection because of more interaction.	Unacast
**Testing (8%)**				
	Daily		*Population divided by tests performed (currently, only state-wide statistics are available).* This is the inverse of the tests per population, so higher numbers indicate less testing. Lower testing rates mean it is more likely that infections are undetected, so this is expected to increase the spread of infection.	The COVID Tracking Project
**Health & Environment (44%)**				
**Population Demographics (8%)**				
**%** Black	Static		*Percentage of the population who self-identify as black or African American.*	2018 "Census Population Estimates" from County Health Rankings and Roadmaps

Data Domain (% weight)<br>Data Slice (% weight)<br>Component(s)	Update Freq.		Description/Rationale	Source(s)
% Native	Static		*Percentage of the population who self-identify as American Indian or Alaska Native.*	2018 "Census Population Estimates" from County Health Rankings and Roadmaps
**Air Pollution (8%)**				
	Static		*Average daily density of fine particulate matter in micrograms per cubic meter ($PM_{2.5}$) from 2014 data from the Environmental Public Health Tracking Network.* Air pollution has been associated with more severe outcomes from COVID-19 infection.	2014 "Environmental Public Health Tracking Network" data
**Age Distribution (8%)**				
**% Age 65 and over**	Static		*Aged 65 or older from the 2014-2018 ACS.* Older age has been associated with more severe outcomes from COVID-19 infection.	2018 CDC Social Vulnerability Index
**Co-morbidities (8%)**				
**Premature death**	Static		*Years of potential life lost before age 75 per 100,000 population (age-adjusted) based on the 2016-2018 National Center for Health Statistics Mortality Files.* This is a broad measure of health and a proxy for cardiovascular and pulmonary disease that has been associated with more severe outcomes from COVID-19 infection.	2020 County Health Rankings
**Smoking**	Static		*Percentage of adults who are current smokers from the 2017 Behavioral Risk Factor Surveillance System.* Smoking has been associated with more severe outcomes from COVID-19 infection and causes cardiovascular and pulmonary disease.	2020 County Health Rankings
**Diabetes**	Static		*Percentage of adults aged 20 and above with diagnosed diabetes from the 2016 United States Diabetes Surveillance System.* Diabetes has been associated with more severe outcomes from COVID-19 infection.	2020 County Health Rankings
**Obesity**	Static		*Percentage of the adult population (age 20 and older) that reports a body mass index (BMI) greater than or equal to 30 kg/m^2.* Obesity has been associated with more severe outcomes from COVID-19 infection.	2020 County Health Rankings

Data Domain (% weight)<br>Data Slice (% weight)<br>Component(s)	Update Freq.		Description/Rationale	Source(s)
**Health disparities (8%)**				
**Uninsured**	Static		*Percentage uninsured in the total civilian noninstitutionalized population estimate, from the 2014-2018 ACS.* Individuals without insurance are more likely to be undercounted in infection statistics and may have more severe outcomes due to lack of treatment.	2018 CDC Social Vulnerability Index (adjunct variable)
**SVI Socioeconomic Status**	Static		*Integrates data from the 2014-2018 ACS on the percentage of residents below the poverty line, the percentage of unemployed residents (historical), income, and percentage of residents without a high school diploma.* Individuals who have lower socioeconomic status are more likely to be undercounted in infection statistics and may have more severe outcomes due to lack of treatment.	2018 CDC Social Vulnerability Index (SVI Socioeconomic Status score)
**Hospital Beds (4%)**				
	Static		*Total hospital beds for hospitals with "OPEN" status and "GENERAL MEDIAL AND SURGICAL" description.*	Homeland Infrastructure Foundation-Level Data (HIFLD)

Table S2. Coefficient table for negative binomial model with cumulative cases up to 8/24/2020 as the response. **P*<0.05 after Bonferroni correction.

Coefficients	Estimate	StdError	Z	P-value
(Intercept)	-6.9370	0.318603962	-21.77	4.18E-105*
AvgSocialDist[a]	0.1526	0.027055473	5.64	1.69E-08*
AvgStatePctTested[a]	4.4429	0.610474548	7.28	3.39E-13*
CHR_Diabetes[a]	0.2774	0.372185894	0.75	4.56E-01
CHR_PrematureDeath[a]	0.0000	0.000007835	-5.22	1.82E-07*
CHR_Smoking[a]	0.8063	0.630131954	1.28	2.01E-01
CHR_Traffic[a]	0.0003	0.000066213	4.84	1.33E-06*
education[a]	0.2771	0.164711964	1.68	9.25E-02
hispanic[b]	1.2419	0.136041709	9.13	6.92E-20*
INSURANCE_PctTot[a]	0.0360	0.003313736	10.88	1.51E-27*
log(SVI_TotPop) [a]	0.9604	0.020324111	47.25	<1E-300*
mean_pm25[b]	0.0961	0.008191268	11.73	9.08E-32*
medhouseholdincome[a]	0.0000	0.000001751	0.03	9.79E-01
medianhousevalue[a]	0.0000	0.000000216	0.89	3.74E-01
pct_asian[b]	-2.2173	0.975900236	-2.27	2.31E-02
pct_blk[b]	1.8332	0.110674804	16.56	1.28E-61*
pct_native[b]	0.7076	0.257704082	2.75	6.04E-03
pct_owner_occ[b]	0.1241	0.196503186	0.63	5.28E-01
popdensity[a]	0.0000	0.000020948	-0.56	5.76E-01
poverty[a]	0.0242	0.266860249	0.09	9.28E-01
q_popdensity[a]	-0.0509	0.017159867	-2.97	3.00E-03
SVI_1_Socioeconomic[a]	0.0005	0.000633677	0.81	4.17E-01
SVI_2_Household[a]	-0.1003	0.062952782	-1.59	1.11E-01
SVI_4_Housing[a]	0.3230	0.056765085	5.69	1.27E-08*
SVI_AreaSqMiles[a]	0.0000	0.000010845	1.89	5.91E-02
SVI_PctGE65[a]	-0.0090	0.004904634	-1.83	6.67E-02
SVI_PctLE17[a]	0.0320	0.005945513	5.38	7.65E-08*
TRAVEL_DaytimePopDensity[a]	0.0000	0.000007292	0.43	6.67E-01

[a] As described in Table S1 and/or the data dictionary on the PVI site.

[b] as used in Wu, X., Nethery, R. C., Sabath, B. M., Braun, D., & Dominici, F. (2020). Exposure to air pollution and COVID-19 mortality in the United States. medRxiv.

Table S3. Coefficient table for negative binomial model with cumulative deaths up to 8/24/2020 as the response. *P<0.05 after Bonferroni correction.(see Table S2 for predictor citations)

**Coefficients**	**Estimate**	**StdError**	**Z**	**P-value**
(Intercept)	-14.0451	0.527123649	-26.64	2.06E-156*
AvgSocialDist[a]	0.0305	0.043557941	0.70	4.84E-01
AvgStatePctTested[a]	5.1067	0.944423358	5.41	6.40E-08*
CHR_Diabetes[a]	0.5790	0.614802450	0.94	3.46E-01
CHR_PrematureDeath[a]	0.0000	0.000013639	0.76	4.50E-01
CHR_Smoking[a]	-1.9581	1.018972398	-1.92	5.47E-02
CHR_Traffic[a]	0.0002	0.000096633	2.39	1.70E-02
education[b]	0.6461	0.272641349	2.37	1.78E-02
hispanic[b]	1.9621	0.219526630	8.94	3.96E-19*
INSURANCE_PctTot[a]	0.0224	0.005496661	4.08	4.48E-05*
log(SVI_TotPop)[a]	1.0203	0.032444802	31.45	4.64E-217*
mean_pm25[b]	0.1356	0.013039105	10.40	2.57E-25*
medhouseholdincome[a]	0.0000	0.000002833	6.70	2.04E-11*
medianhousevalue[a]	0.0000	0.000000360	-1.22	2.22E-01
pct_asian[b]	-3.3992	1.446393084	-2.35	1.88E-02
pct_blk[b]	2.8574	0.170673949	16.74	6.51E-63*
pct_native[b]	2.2643	0.410556502	5.52	3.48E-08*
pct_owner_occ[b]	1.6120	0.322587268	5.00	5.82E-07*
popdensity[b]	0.0001	0.000030450	2.41	1.58E-02
poverty[b]	0.3848	0.447289328	0.86	3.90E-01
q_popdensity[b]	0.0284	0.027567617	1.03	3.04E-01
SVI_1_Socioeconomic[a]	0.0010	0.000951166	1.07	2.83E-01
SVI_2_Household[a]	0.3559	0.104331125	3.41	6.46E-04*
SVI_4_Housing[a]	0.4192	0.091326708	4.59	4.43E-06*
SVI_AreaSqMiles[a]	0.0000	0.000017291	1.27	2.03E-01
SVI_PctGE65[a]	0.0219	0.008130769	2.69	7.05E-03
SVI_PctLE17[a]	0.0114	0.010148466	1.12	2.62E-01
TRAVEL_DaytimePopDensity[a]	0.0000	0.000010533	-2.12	3.40E-02

[a] As described in Table S1 and/or the data dictionary on the PVI site.

[b] as used in Wu, X., Nethery, R. C., Sabath, B. M., Braun, D., & Dominici, F. (2020). Exposure to air pollution and COVID-19 mortality in the United States. medRxiv.

Table S4. Coefficient table for negative binomial model with cumulative death rate (proportion per unit population) up to 8/24/2020 as the response. Accordingly, population is not included as a predictor. **P*<0.05 after Bonferroni correction (see Table S2 for predictor citations).

Coefficients	Estimate	StdError	Z	P-value
(Intercept)	-13.9380	0.491518408	-28.36	6.87E-177*
AvgSocialDist[a]	0.0436	0.038717684	1.13	2.60E-01
AvgStatePctTested[a]	5.1407	0.943389164	5.45	5.06E-08*
CHR_Diabetes[a]	0.5824	0.614492611	0.95	3.43E-01
CHR_PrematureDeath[a]	0.0000	0.000013602	0.73	4.65E-01
CHR_Smoking[a]	-1.9508	1.018618343	-1.92	5.55E-02
CHR_Traffic[a]	0.0002	0.000096173	2.46	1.38E-02*
education[b]	0.6392	0.271704996	2.35	1.86E-02*
hispanic[b]	1.9776	0.217816040	9.08	1.09E-19*
INSURANCE_PctTot[a]	0.0221	0.005474522	4.03	5.59E-05*
log(SVI_TotPop)[a]	0.0000	0.000000000	0.00	0.00
mean_pm25[b]	0.1353	0.013034880	10.38	2.99E-25*
medhouseholdincome[a]	0.0000	0.000002831	6.72	1.77E-11*
medianhousevalue[a]	0.0000	0.000000359	-1.13	2.57E-01
pct_asian[b]	-3.3380	1.441496228	-2.32	2.06E-02
pct_blk[b]	2.8690	0.169575224	16.92	3.27E-64*
pct_native[b]	2.2803	0.409597841	5.57	2.59E-08*
pct_owner_occ[b]	1.6266	0.321477915	5.06	4.20E-07*
popdensity[b]	0.0001	0.000029426	2.67	7.58E-03
poverty[b]	0.3762	0.446996099	0.84	4.00E-01
q_popdensity[b]	0.0362	0.024194081	1.50	1.34E-01
SVI_1_Socioeconomic[a]	0.0010	0.000950496	1.10	2.72E-01
SVI_2_Household[a]	0.3520	0.104116152	3.38	7.24E-04*
SVI_4_Housing[a]	0.4278	0.090029049	4.75	2.01E-06*
SVI_AreaSqMiles[a]	0.0000	0.000016282	1.56	1.20E-01
SVI_PctGE65[a]	0.0220	0.008126560	2.70	6.85E-03
SVI_PctLE17[a]	0.0117	0.010130134	1.15	2.48E-01
TRAVEL_DaytimePopDensity[a]	0.0000	0.000010298	-2.31	2.08E-02

[a] As described in Table S1 and/or the data dictionary on the PVI site.

[b] as used in Wu, X., Nethery, R. C., Sabath, B. M., Braun, D., & Dominici, F. (2020). Exposure to air pollution and COVID-19 mortality in the United States. medRxiv.

Table S5. Coefficient table for negative binomial model with cumulative death rate (proportion among cases) up to 8/24/2020 as the response. **P*<0.05 after Bonferroni correction (see Table S2 for predictor citations).

**Coefficients**	**Estimate**	**StdError**	**Z**	**P-value**
(Intercept)	-6.9978	0.444249920	-15.75	6.67E-56*
AvgSocialDist[a]	-0.1498	0.037551861	-3.99	6.62E-05*
AvgStatePctTested[a]	1.6754	0.800162115	2.09	3.63E-02
CHR_Diabetes[a]	0.1506	0.529860446	0.28	7.76E-01
CHR_PrematureDeath[a]	0.0000	0.000011948	2.88	4.02E-03
CHR_Smoking[a]	-1.6206	0.885719082	-1.83	6.73E-02
CHR_Traffic[a]	0.0000	0.000077170	-0.16	8.75E-01
education[b]	0.2833	0.236841223	1.20	2.32E-01
hispanic[b]	0.7945	0.194019174	4.09	4.22E-05*
INSURANCE_PctTot[a]	-0.0059	0.004787450	-1.23	2.18E-01
log(SVI_TotPop)[a]	0.0660	0.027609236	2.39	1.69E-02
mean_pm25[b]	0.0414	0.011112333	3.73	1.92E-04*
medhouseholdincome[a]	0.0000	0.000002426	7.75	8.90E-15*
medianhousevalue[a]	0.0000	0.000000313	-4.04	5.28E-05*
pct_asian[b]	-1.2215	1.178818629	-1.04	3.00E-01
pct_blk[b]	1.0018	0.143197683	7.00	2.64E-12*
pct_native[b]	1.4255	0.360815341	3.95	7.79E-05*
pct_owner_occ[b]	1.5400	0.276921773	5.56	2.68E-08*
popdensity[b]	0.0001	0.000024531	2.63	8.42E-03
poverty[b]	0.3002	0.390181579	0.77	4.42E-01
q_popdensity[b]	0.0902	0.023504005	3.84	1.24E-04*
SVI_1_Socioeconomic[a]	0.0004	0.000778229	0.48	6.31E-01
SVI_2_Household[a]	0.3054	0.090465880	3.38	7.36E-04*
SVI_4_Housing[a]	0.1827	0.077502418	2.36	1.84E-02
SVI_AreaSqMiles[a]	0.0000	0.000014849	-1.17	2.43E-01
SVI_PctGE65[a]	0.0296	0.006888061	4.29	1.77E-05*
SVI_PctLE17[a]	-0.0266	0.008854467	-3.00	2.67E-03
TRAVEL_DaytimePopDensity[a]	0.0000	0.000008458	-2.10	3.54E-02

[a] As described in Table S1 and/or the data dictionary on the PVI site.

[b] as used in Wu, X., Nethery, R. C., Sabath, B. M., Braun, D., & Dominici, F. (2020). Exposure to air pollution and COVID-19 mortality in the United States. medRxiv.

Table S6. Coefficient table for negative binomial model with daily cases as the response, up to 8/24/2020, using both fixed and dynamic predictors, with standard errors evaluated by bootstrapping. **P*<0.05 after Bonferroni correction (see Table S2 for predictor citations).

**Coefficients**	**Estimate**	**Bootstrap SE**	**Z**	**P-value**
(Intercept)	-14.43413617	0.075410217	-191.408	<1E-300*
AvgSocialDist	0.111212451	0.005620197	19.788	3.78E-87*
AvgStatePctTested	4.881482966	0.118246686	41.2822	<1E-300*
CHR_Diabetes	0.23150235	0.079735564	2.903376	0.003692
CHR_PrematureDeath	-4.17102E-05	1.78482E-06	-23.3694	8.8E-121*
CHR_Smoking	0.981747618	0.129066072	7.606551	2.82E-14*
CHR_Traffic	0.000255246	1.19722E-05	21.31999	7.4E-101*
education	0.251150489	0.035042877	7.166948	7.67E-13*
hispanic	0.951577949	0.0282904	33.63607	5E-248*
INSURANCE_PctTot	0.029098933	0.000704387	41.31101	<1E-300*
log(SVI_TotPop)	0.894560944	0.004162461	214.9115	<1E-300*
mean_pm25	0.079885694	0.001651557	48.36993	<1E-300*
medhouseholdincome	5.83462E-06	3.63979E-07	16.03012	7.87E-58*
medianhousevalue	5.91165E-07	4.34852E-08	13.59464	4.31E-42*
pct_asian	-2.20291288	0.178534374	-12.3389	5.59E-35*
pct_blk	1.878826979	0.02173296	86.45058	<1E-300*
pct_native	0.949723582	0.05231677	18.15333	1.21E-73*
pct_owner_occ	0.484274997	0.040640121	11.91618	9.75E-33*
popdensity	2.06787E-05	4.40212E-06	4.697437	2.63E-06*
poverty	0.252658903	0.056614411	4.462802	8.09E-06*
q_popdensity	-0.024122308	0.003531365	-6.83087	8.44E-12*
SVI_1_Socioeconomic	0.000534244	0.006836522	0.078146	0.937712
SVI_2_Household	-0.096360182	0.013502849	-7.13629	9.59E-13*
SVI_4_Housing	0.447804259	0.011698319	38.27937	<1E-300*
SVI_AreaSqMiles	1.43071E-05	2.1942E-06	6.520421	7.01E-11*
SVI_PctGE65	-0.009289427	0.001034039	-8.98363	2.62E-19*
SVI_PctLE17	0.034249492	0.001267226	27.02715	7.1E-161*
TRAVEL_DaytimePopDensity	-5.92274E-06	2.88391E-06	-2.05372	0.040003
TwoWeekLag	0.002015781	2.97365E-05	67.78814	<1E-300*

Table S7. Coefficient table for negative binomial model with daily deaths/(population size) as the response, up to 8/24/2020, using both fixed and dynamic predictors, with standard errors evaluated by bootstrapping. **P*<0.05 after Bonferroni correction (see Table S2 for predictor citations).

**Coefficients**	**Estimate**	**Bootstrap SE**	**Z**	**P-value**
(Intercept)	-22.52977034	0.295567449	-76.2254	<1E-300*
AvgSocialDist	0.002782565	0.014424908	0.1929	0.847037
AvgStatePctTested	4.689436235	0.257178587	18.23416	2.76E-74*
CHR_Diabetes	0.296758262	0.230684701	1.286424	0.198295
CHR_PrematureDeath	3.61342E-05	4.87903E-06	7.406024	1.3E-13*
CHR_Smoking	-0.245396416	0.319833305	-0.76726	0.442925
CHR_Traffic	-6.41874E-05	2.04256E-05	-3.1425	0.001675*
education	0.916303342	0.093686267	9.780551	1.36E-22*
hispanic	1.239857159	0.069719897	17.7834	9.5E-71*
INSURANCE_PctTot	0.008092203	0.001830856	4.419902	9.87E-06*
log(SVI_TotPop)				
mean_pm25	0.08075838	0.003715508	21.73549	9.5E-105*
medhouseholdincome	2.76163E-05	8.21405E-07	33.62082	8.3E-248*
medianhousevalue	-8.33299E-07	1.00243E-07	-8.31282	9.35E-17*
pct_asian	-3.01631796	0.296529634	-10.1721	2.64E-24*
pct_blk	2.438144797	0.051023053	47.78516	<1E-300*
pct_native	2.353701884	0.119308222	19.72791	1.24E-86*
pct_owner_occ	2.167838189	0.103688442	20.90723	4.6E-97*
popdensity	0.000101865	6.84924E-06	14.87253	4.97E-50*
poverty	0.893419918	0.158632155	5.632023	1.78E-08*
q_popdensity	0.053907171	0.008274928	6.514518	7.29E-11*
SVI_1_Socioeconomic	0.000872714	0.017860144	0.048864	0.961028*
SVI_2_Household	0.211708437	0.03627632	5.835995	5.35E-09*
SVI_4_Housing	0.62565794	0.028576721	21.89397	3E-106*
SVI_AreaSqMiles	6.08249E-06	4.23403E-06	1.436573	0.150839*
SVI_PctGE65	0.044657733	0.002493343	17.91078	9.72E-72*
SVI_PctLE17	0.020277975	0.003478011	5.830336	5.53E-09*
TRAVEL_DaytimePopDensity	-3.04439E-05	4.50973E-06	-6.7507	1.47E-11*
TwoWeekLag	0.001577699	3.72462E-05	42.35864	<1E-300*
